# Case Report: Hidden in motion: a challenging aortic mass unveiled by 3D transesophageal echocardiography

**DOI:** 10.3389/fcvm.2025.1660722

**Published:** 2025-08-29

**Authors:** Mattia Malaguti, Luigi Gerra, Leonardo Fontanesi, Andrea Barbieri, Giuseppe Boriani, Francesca Mantovani

**Affiliations:** ^1^ Cardiology Division, Department of Biomedical, Metabolic and Neural Sciences, University of Modena, and Reggio Emilia, Policlinico di Modena, Modena, Italy; ^2^ Cardiology Division, S. Maria Bianca Hospital, Modena, Italy; ^3^ Cardiology Division, Guglielmo da Saliceto Hospital, Piacenza, Italy; ^4^ Cardiac Surgery and Cardiology Division, Hesperia Hospital, Modena, Italy; ^5^ Division of Cardiology, Azienda USL–IRCCS di Reggio Emilia, Reggio Emilia, Italy

**Keywords:** aortic plaque, atheroma, incidental finding, 3D transesophageal echocardiography, multimodality imaging

## Abstract

This case report describes a 76-year-old asymptomatic male with a highly mobile, elongated, and filamentous structure incidentally detected in the ascending aorta during routine echocardiographic evaluation. The nature of this mass was initially unclear, as its appearance and behavior did not fit the typical features of thrombi, atheroma, vegetations, or dissection. Given the high risk of systemic embolization, timely intervention was deemed necessary. Advanced multimodality imaging, particularly three-dimensional transesophageal echocardiography (3D-TEE), played a pivotal role in defining the lesion's characteristics. Surgical removal was performed, and histopathological analysis provided an unexpected final diagnosis. This case underscores the value of 3D-TEE in assessing rare aortic abnormalities that may not be fully characterized using conventional imaging techniques.

## Graphical Abstract

**Figure:**
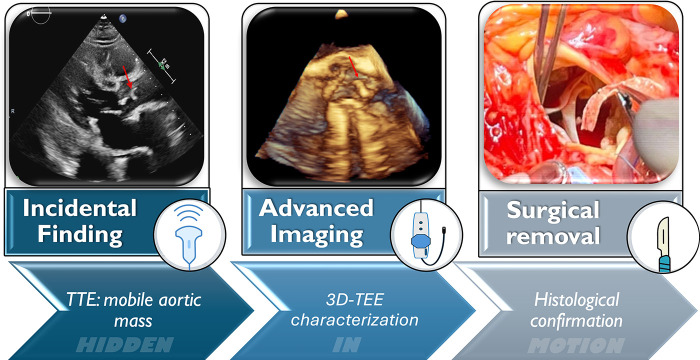
This graphical abstract illustrates the diagnostic pathway of a highly mobile, filamentous mass in the ascending aorta, incidentally found during routine transthoracic echocardiography. 3D transesophageal echocardiography (3D-TEE) played a crucial role in defining the lesion’s morphology and attachment site, leading to surgical excision. Histopathological analysis confirmed a large, calcified atheroma. This case highlights the central role of 3D-TEE imaging in the detection and management of rare aortic abnormalities.

## Introduction

The incidental detection of mobile, strand-like structures in the ascending aorta is rare and presents a significant diagnostic challenge (1). Proper identification is crucial, as management strategies can vary from conservative observation to urgent surgical intervention. Differentiating between thrombus, atheroma, vegetation, and other rare lesions requires a comprehensive approach using multimodality imaging (2).

Transesophageal echocardiography (TEE) is a key imaging tool in assessing intracardiac and aortic abnormalities due to its superior spatial resolution and real-time dynamic imaging capabilities (3). In selected cases, three-dimensional (3D) TEE provides additional insights into lesion morphology and attachment, enabling a more precise characterization that may not be achievable with other imaging modalities.

Here, we present a case of an asymptomatic patient in whom a highly mobile mass was incidentally detected in the ascending aorta. The lesion's unusual characteristics posed a diagnostic dilemma, necessitating advanced imaging and ultimately leading to surgical intervention. Histopathological analysis revealed a surprising diagnosis, highlighting the importance of thorough multimodal evaluation in such cases.

## Case description

A 76-year-old male patient with a history of chronic coronary syndrome was referred for evaluation by routine transthoracic echocardiography. The patient underwent the initial diagnostic workup and part of the management at the Policlinico di Modena, Italy. He had undergone coronary artery bypass grafting ten years earlier. He was affected by several cardiovascular risk factors: arterial hypertension, dyslipidemia, and type 2 diabetes. Moreover, he had stage V chronic kidney disease on peritoneal dialysis. Additionally, he suffered from peripheral artery disease, including prior right renal artery stenosis requiring stenting and non-significant atheromatous disease of the supra-aortic trunks. The family, genetic, and psychosocial histories were unremarkable.

During our evaluation at rest, the patient was asymptomatic from a cardiac standpoint.

### Initial findings

**Figure 1:**
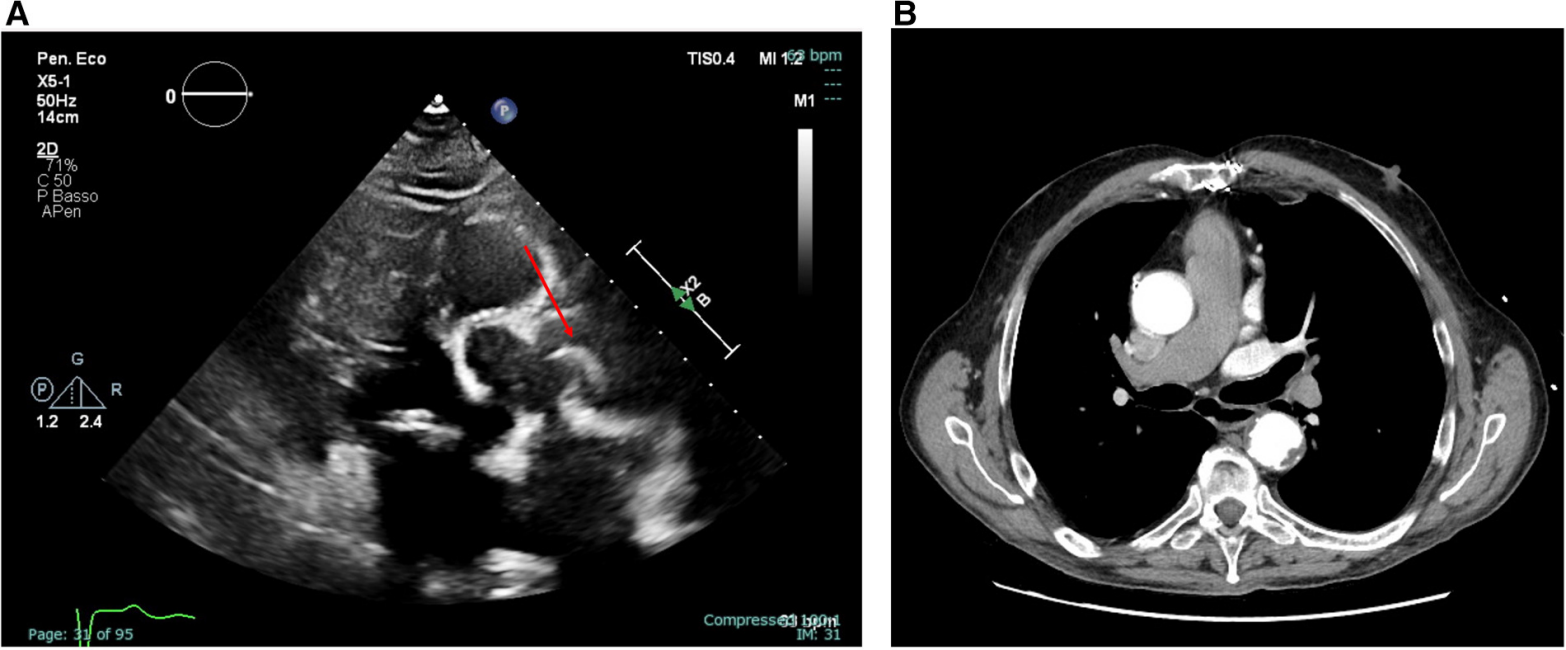
Transthoracic echocardiography (parasternal long-axis view) showing an extremely mobile, strip-shaped filamentous mass located at the level of the sino-tubular junction [**(A)**, left panel, still frame of Supplementary Video S1] and axial view of a thoracic CT angiography not detecting the highly mobile and thin mass [**(B)**, right panel].

The transthoracic echocardiogram revealed an extremely mobile, strip-shaped filamentous mass at the aortic root level above the sino-tubular junction, apparently attached to the aortic wall (Figure 1A- left panel; Supplementary Video S1). The aortic root and ascending aorta were of normal size, and no echocardiographic signs of aortic dissection were present. The exam revealed mild calcification of the aortic valve cusps, accompanied by moderate valvular stenosis. A mildly dilated left ventricle with concentric remodeling and moderately reduced left ventricular ejection fraction was also detected.

### Differential diagnosis

The mass was incidentally found, and the patients denied symptoms possibly related to this finding. However, to exclude possible intimal flap or other acute aortic diseases, the patient was admitted for urgent Cardiac Tomography (CT) angiography, which excluded aortic dissection. Notably, the mass visualized on transthoracic echocardiography was not detectable on CT, despite a thorough review of both contrast-enhanced and non-contrast images (Figure 1B- right panel). As this was a non-gated angiographic CT scan, its sensitivity for detecting small, mobile intraluminal structures was likely limited by motion artefacts and the absence of synchronized acquisition. Indeed, this mass was visualized consistently across multiple echocardiographic windows and probes, ruling out potential artifacts. Blood cultures and laboratory inflammation indices were also negative. Moreover, given the location of the attachment and the morphological characteristics, it was unlikely that the mass represented a lesion of another nature (e.g., Lambl's excrescence or papillary fibroelastoma of the aortic valve).

### Further investigation and diagnosis

**Figure 2:**
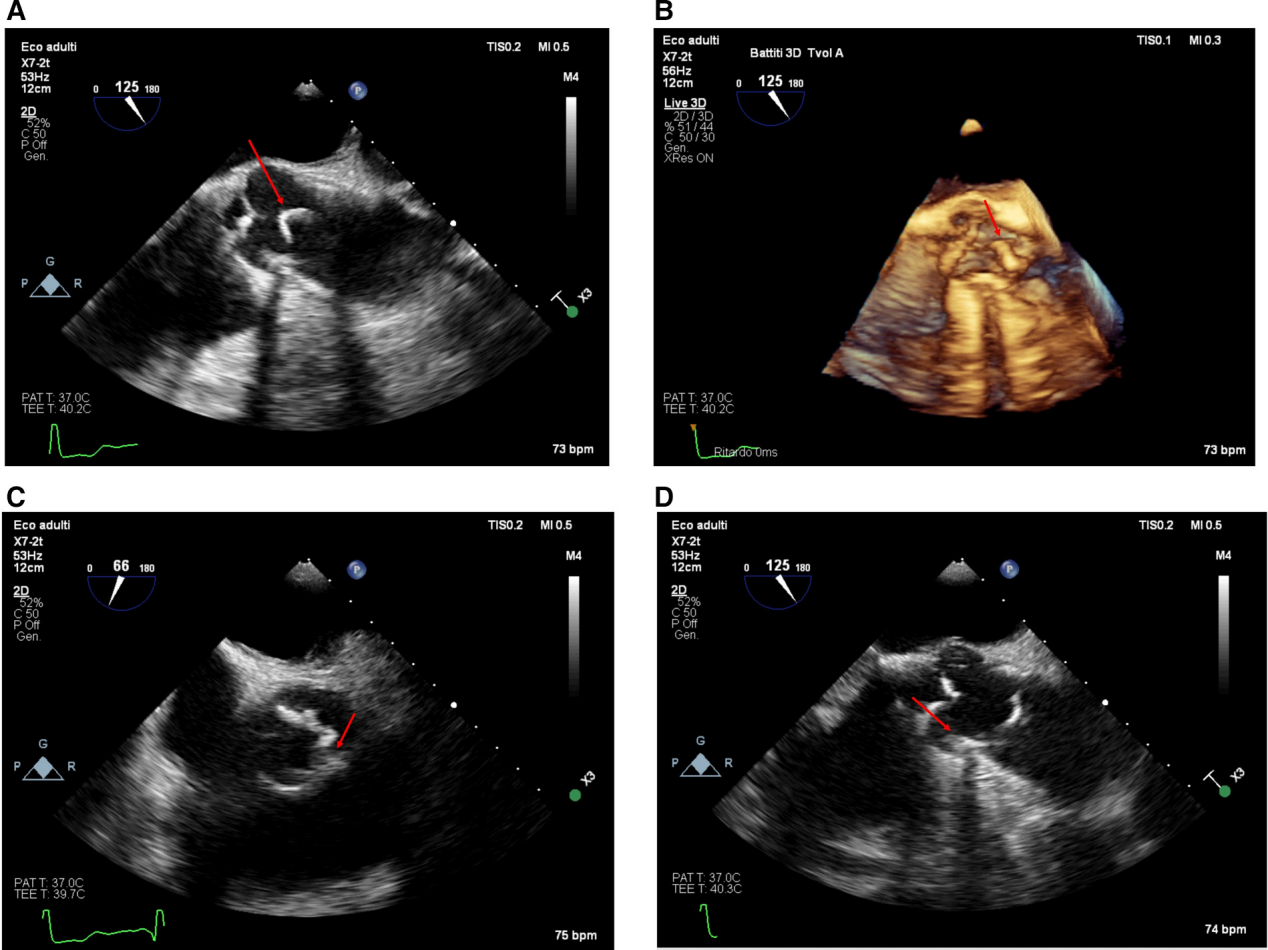
The appearance of the mass from the transesophageal approach at the mid-esophageal 120-degree view (ME AV LAX view) in 2D [**(A)**, upper left panel, taken from Supplementary Video S2] and with a real-time 3D reconstruction [**(B)**, upper right panel, Supplementary Video S3–S4]. The lesion appeared from the transesophageal approach in the mid-esophageal short-axis view at 66° [**(C)**, lower left panel] and the 125° long-axis view [**(D)**, lower right panel]. The calcified base of the plaque is visible (red arrow); notice the shadow cone [**(D)**, lower right panel—red star] arising from the calcified plaque in the long-axis view.

A 3D-TEE was performed to better characterize the lesion (Philips Epiq CVx3D equipped with an X7-2t transesophageal probe). This confirmed a highly mobile, filamentous mass (5 mm × 38 mm) originating from the aortic wall at the level of the sino-tubular junction, attached to a fibrocalcific plaque (Figures 2; Supplementary Video S2). The mass moved with systolic-diastolic excursions without producing obstruction at the valvular level (Supplementary Videos S3–S5). Supplementary Video S6 shows the color X-plane appearance of the lesion in real-time motion.

### Management and surgical intervention

**Figure 3:**
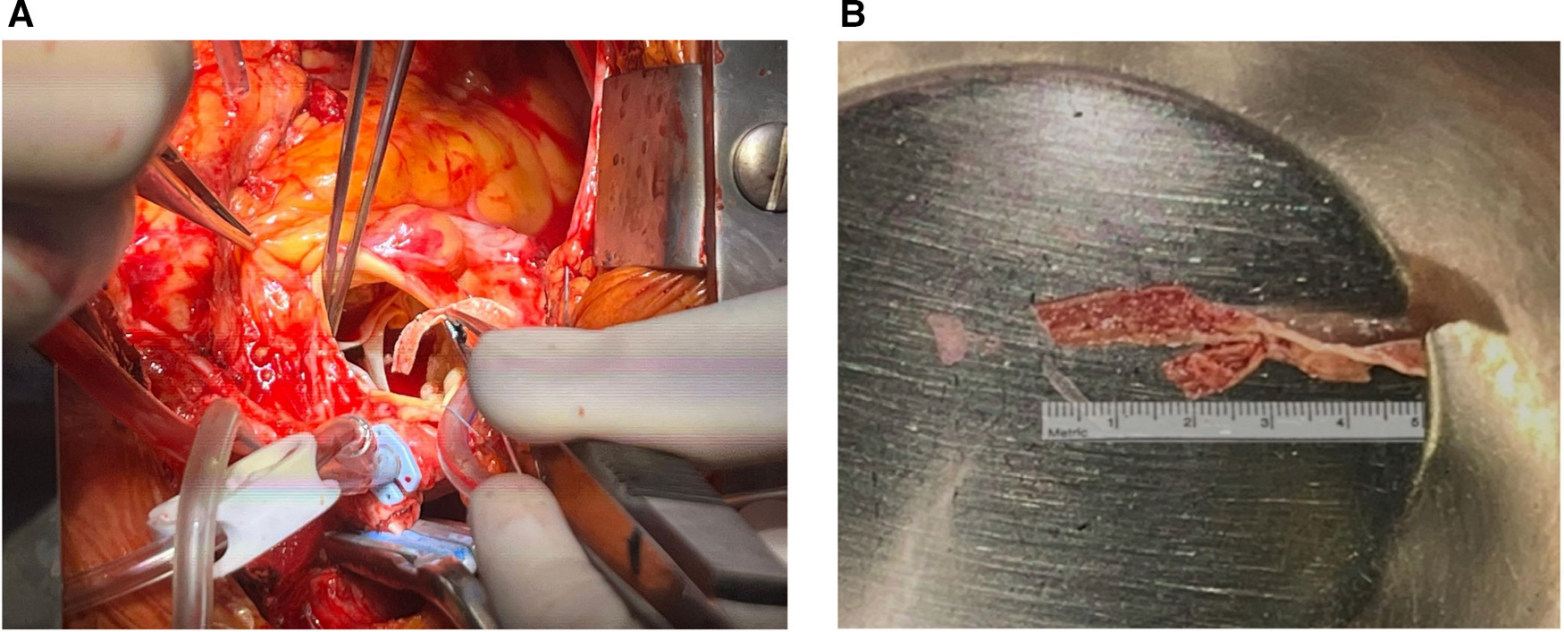
Macroscopic appearance of the surgical specimen during [**(A)**, left panel] and immediately after removal [**(B)**, right panel].

**Figure 4:**
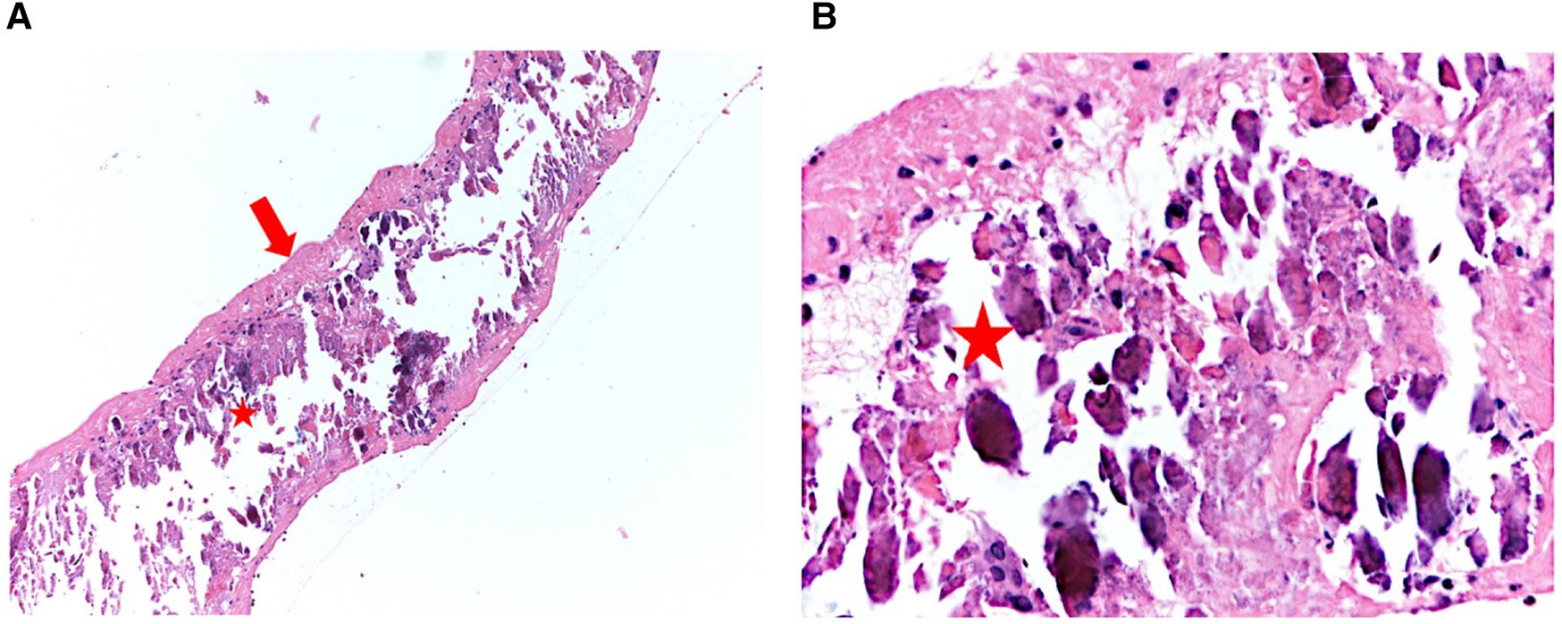
Histopathological analysis of the atheroma demonstrates a thin, fibrous outer layer [**(A)**, left panel—red arrow] with a core abundant in calcium deposits and inflammatory cells (red star). Zoomed image [**(B)**, right panel].

Given the potential diagnosis of a thrombus on an ulcerated aortic plaque, we started unfractionated heparin (with a target aPTT of 1.5–2.3 due to renal failure). After 48 h, no reduction in the mass size was detected by transthoracic echocardiography. Despite the absence of neurological signs and the patient's asymptomatic status, surgical excision was recommended given the high risk of cerebral or systemic embolism. The day after, the patient underwent cardiac surgery. A median sternotomy and transaortic approach allowed the mass to be reached. A strip-shaped 5 cm long fibro-calcific neoformation was attached to the aortic wall at the level of the sino-tubular junction, arising from a calcific plaque (Figures 3A,B). The lesion was quickly removed by pulling its distal extremity with a surgical clamp, confirming the high embolization potential. Aortic valve replacement with a bio-prosthesis (Perceval Plus) was also performed, considering the concomitant moderate aortic stenosis. Histopathological examination of the excised material revealed a thin outer layer of fibrous tissue with a core rich in calcium crystals and inflammatory cells, confirming the diagnosis of a large, mobile, calcified atheroma (Figure 4).

### Outcome and follow-up

The patient experienced an uncomplicated postoperative recovery and was promptly transferred to cardiac rehabilitation for further management. He recovered well during his rehabilitation stay and was discharged after one week in stable condition. The patient expressed satisfaction with the outcome and consented to share his experience for educational purposes.

## Discussion

Large free-floating masses in the ascending aorta are a rare finding. Differential diagnosis is challenging, including penetrating aortic ulcer or atherosclerotic plaques complicated by thrombosis (4–6), infective endocarditis (7) and neoformations at the level of the aortic valve (8). Thrombus formation within the aortic lumen is uncommon due to the high-pressure and high-flow environment of the arterial system. Vegetations associated with infective endocarditis most commonly form on the ventricular side of the aortic valve. Only a few cases of large mobile atheroma of the ascending aorta have been described (9, 10). Moreover, the formation of pedunculated aortic plaques is typically associated with the rupture of soft plaques (11). In our case, histological examination revealed a highly calcified atheroma, making our case even more unique.

Typically, patients affected by high-grade aortic atherosclerosis show several cardiovascular risk factors and comorbidities contributing to a pro-inflammatory state. Indeed, our patient presented with hypertension, diabetes, dyslipidemia, peripheral artery disease, and stage V renal failure, highlighting how these risk factors are also linked to the development of threatening complications of atherosclerotic plaques. In this case, the diagnosis was made incidentally in an otherwise asymptomatic patient. Fortunately, our patient did not have a history of systemic embolism or embolic stroke, although mobile aortic atheroma is frequently associated with embolic episodes (11). Lambl's excrescence, also known as papillary fibroelastoma of the aortic valve, has distinct morphological features compared to our case.

However, among potential differential diagnoses, papillary fibroelastoma (PFE) should also be considered, given its high embolic potential and frequent presentation as a mobile, strand-like mass detected by echocardiography. Although the morphology and anatomical location in our case were not consistent with a typical fibroelastoma, awareness of its varied clinical presentations remains essential (12).

In similar cases where the nature or origin of the lesion remains uncertain, additional imaging modalities such as cardiac magnetic resonance (CMR) may offer further diagnostic insight (13).

CMR is widely regarded as a leading modality for tissue characterization of cardiac masses, enabling detailed differentiation between thrombi, benign tumors, and malignant lesions. CMR remains the preferred technique for characterizing cardiac masses due to its superior soft tissue contrast, allowing differentiation between thrombus, tumor, and artifacts, primarily through the use of T1- and T2-weighted sequences, first-pass perfusion, and late gadolinium enhancement (13). While intracavitary thrombi, such as those in the left ventricle or atrial appendage, are more commonly encountered and better characterized by CMR, masses located in atypical sites like the ascending aorta pose additional diagnostic challenges (14). In these cases, the avascular and non-enhancing nature of thrombi on CMR can still aid in distinguishing them from neoplastic or inflammatory lesions, even when their morphology is unusual. Although thrombus was initially considered, the lesion's firm attachment, heavy calcification, and lack of response to anticoagulation made this hypothesis less likely.

Beyond its diagnostic utility, CMR has also shown prognostic value in patients with suspected cardiac masses.

Recent multicenter data have demonstrated the incremental prognostic value of CMR in patients with suspected cardiac masses, aiding in risk stratification (13, 15). However, in the present case, the heavily calcified and filiform nature of the lesion would likely have limited the diagnostic utility of CMR as well. This, combined with the fact that transesophageal echocardiography had already provided sufficient morphological and dynamic information to support a working diagnosis and guide clinical decision-making, and considering the time-sensitive logistics of CMR acquisition and reporting in the acute care setting, led us to defer further imaging reasonably.

Interestingly, this highly mobile pedunculated lesion was not identified on CT angiography. The scan was performed without ECG gating, as per standard protocol for urgent aortic angiographic studies, which may have limited the detection of small, fast-moving structures due to pulsation artefacts. Moreover, the mass was not visible on the non-contrast CT images obtained before contrast injection. Despite its considerable length (approximately 5 cm), the lesion was extremely thin and calcified, with a morphology likely falling below the spatial resolution threshold of the scanner (0.23 mm). Additionally, its detection may have required a non-standard imaging plane to capture both the site of attachment and its intraluminal course. In contrast, transthoracic echocardiography, performed using multiple probes and acoustic windows, offered superior temporal resolution, enabling the visualization of the mass's mobility and fine structure, which ultimately supported the clinical decision-making process. The TEE imaging was crucial for the final diagnosis, demonstrating that the formation was a component of a more extensive, intricate, and diffuse process that was not readily evident on 2D imaging, protruding from the aorta surface in conjunction with a localized thickening. These results suggest a connection with the intima, possibly due to atherogenesis or damage that disrupts or lifts the intimal surface (16, 17).

Nevertheless, once the diagnostic process was concluded, the therapeutic approach remained challenging, as management of such lesions is still controversial and must be tailored to the individual patient's risk profile and comorbidities (5, 18). Of note, observational studies demonstrated that 20%–30% of patients with embolic strokes might have aortic atheroma on TEE (19). In these patients, thrombi probably develop on plaques and cause embolic events; however, when the plaque burden is high and the plaques are heavily calcified, as in our case, there might not be a response to anticoagulation therapy. In our patient, considering the high embolic risk and the lack of response to anticoagulation therapy, we recommended surgical intervention, although the patient was asymptomatic.

## Conclusions

This case underscores the importance of 3D-TEE in detecting and characterizing rare, highly mobile aortic lesions that may not be visualized on other imaging modalities. The incidental finding of a floating mass in the ascending aorta requires a thorough diagnostic approach, given the potential for catastrophic embolic complications. In cases where anticoagulation is ineffective or when the embolic risk is high, surgical intervention should be strongly considered (9).

## Data Availability

The original contributions presented in the study are included in the article/Supplementary Material, further inquiries can be directed to the corresponding author.

## Supplementary material

Supplementary Video 1 Transthoracic echocardiography (parasternal long-axis view) showing a highly mobile filamentous mass. This video corresponds to Figure 1 A (left panel).

Supplementary Video 2 2D transesophageal echocardiography (TEE) in the mid-esophageal 120° long-axis view, depicting the mass and its attachment. This video corresponds to Figure 2 A, upper left panel.

Supplementary Video 3-4 Real-time 3D-TEE reconstructions illustrating the mass's morphology and motion (Figure 2 B, upper right panel).

Supplementary Video 5 TEE short-axis view (66°), displaying the lesion and its calcified base.

Supplementary Video 6 Color Doppler X-plane view of the mass in real-time motion.
